# Clinical trial registration: internal conflict?

**DOI:** 10.3325/cmj.2011.52.745

**Published:** 2011-12

**Authors:** Farrokh Habibzadeh

**To the Editor:** To assure transparency and reporting of all conducted clinical trials, in 2004, the International Committee of Medical Journal Editors (ICMJE) announced its statement on clinical trial registration (1). According to this statement, any clinical trial should be registered before its beginning in certain registries, so that researchers can be aware of its progress and results at different stages of the study. This statement was soon endorsed by many influential organizations and many journals have asked for mandatory registration of trials and reporting the registration number of any submitted trial prior to its evaluation and peer review. Even many small medical journals abide to this rule (2). Previously, I discussed the impact of mandatory clinical trial registration on small medical journals (3). Now, I would like to assess the internal consistency of this statement through an example.

**Figure 1:**
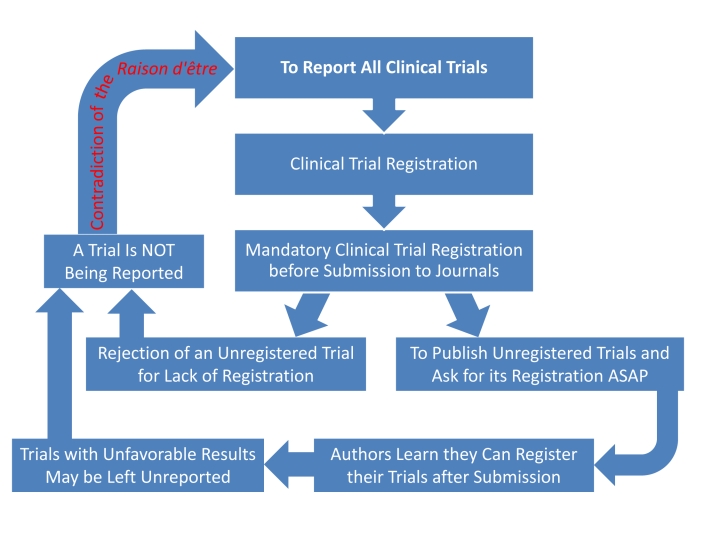
Schematic diagram depicting the internal conflict in Clinical Trial Registration Policy.

Assume that you are the editor of a journal adhering to the trial registration statement and receive a clinical trial that has not been registered. What will you do? According to the rules set, you should reject the manuscript on the fly and not send it for peer review as it has not been registered. But, let us examine the *raison d'ętre* of establishing this statement – to report all clinical trials conducted (assume that the trial is methodologically acceptable). Therefore, if you do not let the said manuscript being peer reviewed and published, you contradict the very basic reason why the statement exists. Therefore, to abide to the statement, ie, to publish all conducted clinical trials, you should let the manuscript be reviewed but ask the author(s) to register it as soon as possible in an acceptable registry. But, if researchers learn that they can postpone registration of their trials to the time after submission, then only those trials with favorable results might be registered and reported and this clearly contradicts the *raison d'ętre* of the trial registration statement again. Therefore, it seems that no matter what option you choose, ie, to accept or not to accept submission of an unregistered clinical trial, you violate the *raison d'ętre* of this statement and that is why I believe this statement is self-contradictory and we have to think about another approach to assure reporting of all clinical trials (Figure 1).
